# Epidemiology of Drug- and Herb-Induced Liver Injury Assessed for Causality Using the Updated RUCAM in Two Hospitals from China

**DOI:** 10.1155/2021/8894498

**Published:** 2021-02-24

**Authors:** Yongwu Chen, Chongwei Wang, Hui Yang, Ping Huang, Jiana Shi, Yongxi Tong, Jinying Jiang, Xin Zhang, Wanyuan Chen, Zixue Xuan

**Affiliations:** ^1^Department of Pharmacy, The First Affiliated Hospital of USTC, Division of Life Sciences and Medicine, University of Science and Technology of China, Hefei 230036, China; ^2^Office of Drug Clinical Trial Institution, The First Affiliated Hospital of USTC, Division of Life Sciences and Medicine, University of Science and Technology of China, Hefei 230036, China; ^3^Department of Pharmacy, Zhejiang Provincial People's Hospital, People's Hospital of Hangzhou Medical College, Hangzhou, China; ^4^Departments of Infection Diseases, Zhejiang Provincial People's Hospital, People's Hospital of Hangzhou Medical College, Hangzhou, China; ^5^Department of Pathology, Zhejiang Provincial People's Hospital, People's Hospital of Hangzhou Medical College, Hangzhou, China

## Abstract

Drug- and herb-induced liver injury (DILI and HILI) is an increasingly common and serious condition. Here, data for DILI and HILI patients from two large tertiary hospitals were retrospectively analyzed. Patient characteristics, causes and severity of DILI and HILI, the correlation between expression of p62 and the severity of DILI and HILI, treatment of DILI and HILI, and the prognostic factors of DILI and HILI were studied. A total of 82 patients with DILI and HILI were recruited for the study. Most patients presented with hepatocellular injury, followed by cholestatic injury and mixed injury. Our results indicate that traditional Chinese medicine or herbal and dietary supplements were the prevalent causal agents of HILI, which was characterized by higher frequencies of hepatocellular injury. Expression of p62 in the liver correlated with the severity of DILI and HILI. Improvements in the results of the liver enzymatic tests correlated with alanine transaminase (ALT) levels upon the first diagnosis of DILI and HILI and with the hepatocellular type of DILI and HILI. In conclusion, we provide an epidemiological assessment of DILI and HILI based on causality using the updated RUCAM on patients from two hospitals in China. ALT levels at first diagnosis and the hepatocellular type of injury may be prognostic factors of DILI and HILI.

## 1. Introduction

Drug- and herb-induced liver injury (DILI and HILI) is an important cause of hepatic failure and can even progress to death [1, 2]. Many studies indicate that the leading causes of herb-induced liver injury (HILI) in China are traditional Chinese medicine (TCM) and herbal and dietary supplements (HDS) [3, 4]. To assess the current status of DILI and HILI, we evaluated DILI and HILI cases recorded between October 2018 and March 2020 from two large tertiary hospitals in China.

First, this study sought to obtain an overview of patient characteristics, as well as causes, type, and severity of DILI and HILI. Second, after DILI and HILI cases were assessed using the updated Roussel Uclaf Causality Assessment Method (RUCAM), liver expression of p62 in DILI and HILI patients was investigated. The multifunctional, stress-induced p62 (SQSTM1) scaffold protein is involved in several cellular processes, including autophagic clearance, regulation of inflammatory responses, and redox homeostasis. Importantly, p62 has been associated with DILI and HILI, although its expression [5–7] and correlation with the type of DILI and HILI, causative drugs, and severity has not been clarified [8]. The present study investigated this correlation. Lastly, we analyzed the association between clinical characteristics and the prognosis of DILI and HILI, which was evaluated by assessing liver test values after treatment with anti-inflammatory and hepatoprotective agents (AIHPAs).

## 2. Materials and Methods

### 2.1. Subjects

This retrospective study was approved by the ethics committees of Zhejiang Provincial People's Hospital and the First Affiliated Hospital of the University of Science and Technology of China. Patients included in our study were hospitalized in the above two hospitals with a diagnosis of DILI and HILI between October 2018 and March 2020.

The inclusion criteria were as follows: clear diagnosis of DILI and HILI, updated RUCAM score ≥ 6 for every case (the correlation between the suspected drug(s) and liver injury is classified as “highly probable” (≥9 points), “probable” (6–8 points), “possible” (3–5 points), “unlikely” (1–2 points), and “excluded” (≤0 points)) [9, 10]; complete records of the gender, age, causal agents, pharmacological treatment, and other information of patients; complete records of the laboratory tests when DILI and HILI was first diagnosed, including levels of aspartate aminotransferase (AST), alanine transaminase (ALT), alkaline phosphatase (ALP), gamma-glutamyl transpeptidase (GGT), and total bilirubin (TBIL), as well as complete records of laboratory tests after 1 week (±2 days) of AIHPA treatment. Informed consent was not required from patients owing to the retrospective nature of this study.

The exclusion criteria were as follows: updated RUCAM score < 6 for every case; laboratory examination findings of patients not meeting the diagnostic criteria for DILI and HILI [9, 10].

### 2.2. Definition and Classification of DILI and HILI

According to the updated RUCAM [9], the type of DILI and HILI was classified by the *R* value calculated from the laboratory data obtained at presentation: *R* value = serum [ALT/ALT ULN]/[ALP/ALP ULN], where ULN is defined as the upper limit of normal [11]. Accordingly, DILI and HILI cases were clinically divided into the following: (1) hepatocellular injury: ALT ≥ 5 × ULN and *R* value ≥ 5; (2) cholestatic injury: ALP ≥ 2 × ULN and *R* value ≤ 2; and (3) mixed injury: ALT ≥ 3 × ULN, ALP ≥ 2 × ULN, and 2 < *R* value < 5. Because liver enzyme levels vary with disease progression, the type of DILI and HILI was determined based on laboratory data when DILI and HILI was first diagnosed.

### 2.3. Etiology of Liver Injury Cases

Causal agents obtained from the hospital medical records were classified as follows: medication, TCM, HDS, and others. The medication category was subdivided according to the affected organ system or the mechanism of action. HDS included vitamins, amino acids, and other nutrients.

### 2.4. Severity of DILI and HILI

The severity of DILI and HILI was determined according to the Drug-Induced Liver Injury Network (DILIN) severity score as follows: mild DILI and HILI (grade 1): raised ALT or/and ALP levels, but TBIL level < 2.5 ULN and international normalized ratio (INR) <1.5; moderate DILI and HILI (grade 2): raised ALT or/and ALP levels, and TBIL level > 2.5 ULN or INR > 1.5 without raised TBIL; moderate to severe DILI and HILI (grade 3): raised ALT or/and ALP levels, and TBIL level > 5 ULN and hospitalization (or prolonged preexisting hospitalization); severe DILI and HILI (grade 4): raised ALT or/and ALP levels, TBIL > 10 ULN, INR ≥ 2 or plasma thromboplastin antecedent (PTA) <40%, and at least 1 of the following: (1) prolonged jaundice and symptoms beyond 3 months, (2) signs of hepatic decompensation (INR > 1.5, ascites, encephalopathy), or (3) other organ failure believed to be related to DILI and HILI.

### 2.5. Histological Analysis and Immunohistochemistry of p62

Liver biopsies were performed, liver tissues were fixed in 10% formalin, and the sections were stained using hematoxylin-eosin plus Sirius red [12]. For the immunohistochemical (IHC) staining of p62, liver sections were first blocked with goat serum, incubated overnight at 4°C with anti-p62 antibody (1 : 100, ab207305; Abcam, Cambridge, UK), and then with horseradish peroxidase-conjugated goat anti-rabbit IgG H and L (1 : 2000; ab205718; Abcam) [6]. The expression of p62 was scored by two pathologists independently using light microscopy, based on the intensity and the proportion of positively stained cells. Signal intensity was evaluated according to the following grading system: 0, negative; 1, weak; 2, moderate; and 3, strong. The percentage of positive cells was scored as follows: 0, <5%; 1, 1–25%; 2, 26–50%; 3, 51–75%; 4, >75% of cells stained. Lastly, scores for intensity and percentage were multiplied [13].

### 2.6. Prognostic Factors of DILI and HILI

To reveal the prognostic factors of DILI and HILI, we assessed the levels of liver enzymes after 1 week (±2 days) of treatment with AIHPAs in all patients with DILI and HILI [14]. An improvement of ≥50% compared to the baseline indicated a reduced level of ALT in hepatocellular injury, a reduced level of ALP in cholestatic injury, and a reduced level of both ALT and ALP in mixed injury.

### 2.7. Statistical Analysis

All statistical analyses were conducted using SPSS 24.0 (SPSS Statistics for Windows, Version 24.0. IBM Corp., Armonk, NY), and data are presented as the mean ± SD. A *t*-test was used for comparison of normal distribution and homogeneity of variance. Nonnormally distributed parameters were compared using the Mann-Whitney *U* test. Categorical variables were compared using Pearson's *χ*^2^ test or Fisher's test. The Cox regression model was used to determine the truncation value of continuous variables. Univariate and multivariate logistic regression analyses were performed and the univariate regression *p* value < 0.1 included multivariate regression. A *p* value < 0.05 was considered as indicating statistical significance.

## 3. Results and Discussion

### 3.1. Characteristics of DILI and HILI Patients

A total of 82 patients were included in the study. Characteristics of DILI and HILI patients including demographic, clinical, and laboratory variables are summarized in Table 1. Result shown that there was no significant difference of age and gender between HILI and DILI, serum ALT levels of HILI increased more significantly than that of DILI (*p* = 0.002).

### 3.2. Causes of DILI and HILI

In this study, the most common causative agent of DILI and HILI was medication (52; 63.41%), including 18 DILI cases caused by antitumor agents (21.95%), 11 by antimicrobial agents (13.41%), 4 by analgesic-antipyretic agents (4.88%), and 19 by other drugs (data not shown). In addition, there were 30 HILI caused by TCM or HDS (30; 36.59%). Single classes of causal agents resulting in the occurrence of DILI of >1% included cardiovascular drugs (7.32%), hormones (3.66%), antihyperthyroidism drugs (2.44%), drugs used for musculoskeletal disorders (2.44%), and psychotropics (1.22%) (data not shown).

HILI was mainly of the hepatocellular type (86.67%), whereas cholestatic and mixed type injuries presented a much lower incidence (6.67% each), indicating HILI is more likely to lead to hepatocellular injury type liver damage. DILI is more prone to cholestatic type liver damage (*p* = 0.001) (Table 2). DILI induced by antitumor drugs was mainly of the cholestatic type (61.11%), although it caused also hepatocellular-type DILI (38.89%). Lastly, 63.64% of DILI caused by antimicrobial drugs was of the hepatocellular type, while the use of analgesic-antipyretic drugs led to various topologies of DILI. However, the sample size is small, which is worthy of further analysis.

### 3.3. Severity of DILI and HILI

The severity of DILI and HILI was assessed based on the DILIN severity score. Out of a total of 81 DILI and HILI patients (no TBIL data were available in 1 case), DILI and HILI severity was of grade 1 for 51 patients, grade 2 for 13 patients, grade 3 for 11 patients, and grade 4 for 6 patients, and the severity of HILI was higher than that of DILI (*p* = 0.008) (Table 1).

### 3.4. Correlation between Expression of p62 and the Severity of DILI and HILI

**Figure 1:**
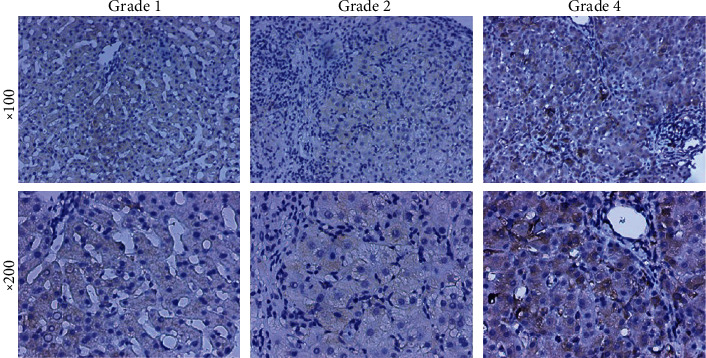
Correlation between the expression of p62 and the severity of DILI&HILI.

We found that p62 was positively expressed in the liver of most patients with DILI and HILI and that the IHC score of p62 correlated with the severity of DILI and HILI (*p* = 0.004, Table 3). Specifically, a high p62 score often indicated a high grade of DILI and HILI severity (Figure 1). In contrast, there was no correlation between the expression of p62 and gender, age, type of liver damage, or causative pharmacological treatment (data not shown).

**Figure 2:**
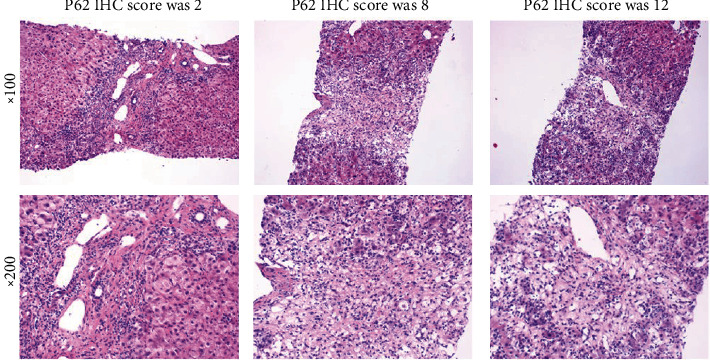
Pathological features were associated with the expression of p62.

Although there is only a limited correlation between biochemical categorization and pathological type of injury [15], focal necrosis can be seen in mild lobular hepatitis, whereas fused necrosis of reticular stent staining (sometimes with bridging necrosis zones) can be seen in moderate lobular hepatitis, characterizing acute DILI and HILI with hepatocellular injury. In severe cases, there is significant bridging necrosis or multilobular necrosis. In the present study, we explored the correlation between p62 expression and severity of DILI and HILI as determined based on the liver-biopsy findings of 14 patients. Among these patients, the injury was of the hepatocellular type in 9 cases, cholestatic type in 4 cases, and mixed type in 1 case. Histological examination revealed that patients with hepatocellular type of DILI and HILI exhibited a higher degree of inflammation, necrosis, and apoptosis. An elevated p62 score was associated with significant bridging necrosis or multilobular necrosis, whereas a low p62 score was associated with mild lobular hepatitis with focal necrosis (Figure 2).

### 3.5. Independent Factors of Prognosis

During their hospitalization, patients were treated with AIHPAs, including glycyrrhizic acid preparations, dicyclool, reductive glutathione, adenosine methionine, ursodeoxycholic acid, polyene phosphatidylcholine, silybin, and other hepatoprotective agents. However, their treatment regimens were different; 8 patients received monotherapy, 26 received a combination of two AIHPAs, and 48 received a combination of three or more AIHPAs (Table 1).

In this study, we evaluated the prognosis of patients with DILI and HILI by comparing ≥50% and <50% improvements in liver test results after 1 week (±2 days) of AIHPA treatment. Univariate analysis revealed significant improvements in relation to the first set of ALT and ALP values, as well as types of DILI and HILI (Table 4).

Based on multivariate analysis, patients whose first ALT was ≥414.5 IU/L had an odds ratio (OR) of 20.651 (95% confidence interval (CI), 2.208–193.099; *p* = 0.008) and those with hepatocellular type had an OR of 26.337 (95% CI, 1.563–443.648; *p* = 0.023) compared to those with the mixed type (Table 5). These results indicated that an initial value of ALT ≥ 414.5 IU/L and hepatocellular type of DILI and HILI were independent factors causing ≥50% improvement in liver tests after 1 week (±2 days) of AIHPA treatment.

The liver is an important organ for drug metabolism and transformation as well as for overall metabolism and immunity. The incidence of DILI and HILI has seen a steady increase over recent years, and DILI and HILI has become a common and serious drug-induced condition worldwide [16, 17].

Here, we analyzed DILI and HILI cases between October 2018 and March 2020 from two large tertiary hospitals. We found that 62.20% of 82 cases with the hepatocellular type met the threshold of “Hy's Law.” Consistent with previous studies in China [18], the present findings revealed that 36.59% were HILI. These results suggested that TCM or HDS were responsible for a high incidence of liver damage and was in agreement with previous reports [19]. The use of TCM or HDS in China has a long history. Both TCM and HDS consist largely of natural components and are associated with few adverse reactions and negligible toxicity [20], although no in-depth study on the toxicology of TCM or HDS has been performed. Evidence points to a complex formulation for TCM or HDS, whose ingredients not only exert a therapeutic effect but can also result in known and unknown adverse reactions [21]. Therefore, detailed analyses of TCM are essential and urgent to determine the presence of toxic ingredients. In line with previous reports [18, 22], we found that the rate of DILI caused by antitumor or antimicrobial agents was only slightly lower than that of TCM or HDS, whereas analgesic-antipyretic agents were another common trigger of DILI. A very similar gender distribution ratio among patients with DILI and HILI (53.66% for men vs. 46.34% for women, *p* > 0.05) was found in our study, suggesting that susceptibility was not affected by gender [23].

HILI presented with higher frequency of hepatocellular injury, whereas DILI is more prone to cholestatic type liver damage, and analgesic-antipyretic is more likely to lead to mixed type liver damage. Additionally, 20.99% of patients were classified as presenting DILI and HILI with severity of grades 3–4, and the severity of HILI was higher than that of DILI. To determine whether the expression of p62 in DILI and HILI tissue was related to the type of DILI and HILI as well as to the type of causative drugs and severity, we analyzed the IHC results of p62 in liver biopsies. We found that p62 was positively expressed in the liver of most patients with DILI and HILI and that the IHC score of p62 correlated with the severity of DILI and HILI. These findings indicated that p62 played an important role in DILI and HILI, especially in evaluating its severity. Previously, p62 was shown to colocalize with acetaminophen (APAP) in primary mouse hepatocytes treated with the drug [8]. Moreover, p62-depleted hepatocytes manifested lower APAP clearance and increased necrosis compared to p62-expressing hepatocytes after APAP treatment [8, 24].

AIHPA treatment regimens during hospitalization differed among patients with some of them receiving monotherapy, while others received a combination of two or more AIHPAs. Laboratory tests that were performed upon the first diagnosis of DILI and HILI indicated that ALT levels, ALP levels, and type of DILI and HILI led to significantly different improvements in liver tests (≥50% and <50%) after 1 week (±2 days) of AIHPA treatment. In particular, multivariate analysis indicated that an initial ALT value of ≥414.5 IU/L and hepatocellular type of DILI and HILI were independent factors of prognosis. The findings also showed that the combined use of two or more types of AIHPAs did not improve the therapeutic efficacy of DILI and HILI, thus corroborating the lack of guidelines in favor of combination therapies. This result is consistent with CSH guidelines for the diagnosis and treatment of DILI and HILI [25]. Additionally, because some AIHPAs may further increase the burden on the liver, we suggest that single AIHPA should be selected for therapy according to the type of DILI and HILI.

## 4. Conclusions

The findings of our study suggest that HILI is related to a higher frequency with hepatocellular injury and that the IHC score of p62 is correlated with the severity of DILI and HILI. We also found that improvements in the findings from the liver tests were related to ALT measurements at the time of the first diagnosis and to the hepatocellular type of DILI and HILI rather than to the combination of AIHPAs. Nevertheless, there are also some limitations to this retrospective study, such as the inevitable existence of bias and the small number of liver biopsies performed. Therefore, a prospective DILI and HILI cohort study may provide a better understanding of DILI and HILI in China. In addition, the role and mechanism of p62 and other autophagy-related genes in DILI and HILI should be explored in future research.

## Figures and Tables

**Table 1:** Clinical, etiologic, and laboratory parameters of the 82 cases of DILI and HILI.

Variable	HILI (*N* = 30)	DILI (*N* = 52)	*χ* ^2^/H	*p*
Age				
≥49	15	29	0.255	0.651
<49	15	23		
Gender				
Male	14	30	0.930	0.335
Female	16	22		
Laboratory findings				
ALT ≥ 5 × ULN	28	32	9.783	0.002
ALT ≥ 3 × ULN and <5 × ULN	1	6		
ALT < 3 × ULN	1	14		
Severity				
Grade 1	14	37	6.984	0.008
Grade 2	6	7		
Grade 3	4	7		
Grade 4	6	0		

**Table 2:** The relationship between the types of DILI and HILI and causal agents.

Types	Hepatocellular	Cholestatic	Mixed	*χ* ^2^	*p*
HILI	26 (3.5^∗^)	2 (-3.4)	2 (-0.5)	14.875	0.001
DILI	25 (-3.5)	22 (3.4)	5 (0.5)		
Most common agents implicated of DILI (Top3)					
Antitumor	7 (-0.8)	11 (2.0)	0 (-2.0)	9.094	0.031
Antimicrobial	7 (1.5)	3 (-1.5)	1 (0.0)		
Analgesic-antipyretic	1 (-0.9)	1 (-0.9)	2 (3.0)		

^∗^Adjusted residuals appear in parentheses below observed frequencies.

**Table 3:** Linear regression of p62 IHC score and grade of DILI and HILI severity.

Serial number	1	2	3	4	5	6	7	8	9	10	11	12	13	14	*p*
Grade of DILI and HILI severity	1	1	1	1	4	2	1	3	1	3	1	1	4	3	0.004
P62 IHC score	8	0	8	8	12	8	8	8	8	12	3	2	12	12	

**Table 4:** Comparison of liver tests improvement ≥50% and <50% after 1 week (±2 days) of AIHPAs treatment.

Variable (mean ± SD, *n*(%))	Liver tests improvement after 1 week (±2 days) AIHPAs treatment		*t*/*U*/*χ*^2^	*p*
	≥50%	<50%		
Age (years)	47.33 ± 13.69	51.21 ± 16.17	-1.170	0.245
The first set of ALT level (IU/L)	795.58 ± 644.66	256.26 ± 211.60	237.500	≤0.001
The first set of ALP level (IU/L)	185.55 ± 124.16	401.51 ± 294.86	1277.500	≤0.001
The first set of TBIL level (*μ*mol/L)	82.26 ± 108.19	57.56 ± 69.99	672.500	0.226
Gender				
Male	26 (31.70)	18 (21.95)	0.012	0.913
Female	22 (26.83)	16 (19.51)		
Types				
Hepatocellular	44 (53.66)	7 (8.54)	46.653	≤0.001
Cholestatic	3 (3.66)	21 (25.61)		
Mixed	1 (1.22)	6 (7.32)		
Severity of DILI and HILI				
Grade 1	28 (34.57)	23 (28.40)	0.560	0.454
Grade 2	9 (11.11)	4 (4.94)		
Grade 3	5 (6.17)	6 (7.41)		
Grade 4	5 (6.17)	1 (1.23)		
Treatment by AIHPAs				
Monotherapy	5 (6.10)	3 (3.66)	0.746	0.388
Combination with 2 drugs	12 (14.63)	14 (17.07)		
Combination with drugs ≥3	31 (37.80)	17 (20.73)		

**Table 5:** Multivariate logistic regression analysis of liver tests improvement ≥50% after 1-week (±2 days) of AIHPAs treatment.

Risk factors	*β*	S.E	Wald	*p*	OR (95% CI)
The first set of ALP ≤ 219 IU/L	1.726	1.517	1.294	0.255	5.618 (0.287~109.955)
The first set of ALT ≥ 414.5 IU/L	3.028	1.141	7.047	0.008	20.651 (2.208~193.099)
^∗^Type of DILI and HILI (1)	3.271	1.441	5.154	0.023	26.337 (1.563~443.648)
^∗^Type of DILI and HILI (2)	2.142	1.640	1.704	0.192	8.513 (0.342~212.042)

^∗^The type of DILI and HILI (1) represented hepatocellular type, and the type of DILI and HILI (2) represented cholestatic type, and all were compared with mixed type.

## Data Availability

The data used to support the findings of this study are available from the article.
